# Enhanced IL36RN Expression and Its Association With Immune Microenvironment Predicts Poor Prognosis in Gastric Cancer

**DOI:** 10.1002/cam4.70954

**Published:** 2025-05-10

**Authors:** Xiaojing Zhang, Sutian Jiang, Hang Yin, Hui Zhang, Lei Yang, Pingping Sun, Xiaoling Kuai, Chen Chen, Jianfei Huang

**Affiliations:** ^1^ Clinical and Translational Research Center, Affiliated Hospital of Nantong University and Medical School of Nantong University Nantong Jiangsu China; ^2^ Department of Clinical Biobank and Institute of Oncology Affiliated Hospital of Nantong University Nantong Jiangsu China; ^3^ Department of Pathology Lishui People's Hospital Lishui Zhejiang China; ^4^ Department of Gastroenterology Nantong University Affiliated Hospital Nantong Jiangsu China; ^5^ Department of Oncology Jiangsu Cancer Hospital and Jiangsu Institute of Cancer Research and the Affiliated Cancer Hospital of Nanjing Medical University Nanjing Jiangsu China; ^6^ The Comprehensive Cancer Centre of Nanjing Drum Tower Hospital, the Affiliated Hospital of Nanjing University Medical School Nanjing Jiangsu China

**Keywords:** CD8+ T cells, gastric cancer, IL36RN, prognosis, tumor microenvironment

## Abstract

**Background:**

Gastric cancer (GC) remains a prevalent and lethal malignancy worldwide, underscoring the urgent need to identify novel therapeutic targets and elucidate the tumor microenvironment (TME) to enhance clinical outcomes.

**Methods:**

IL36RN mRNA expression in GC tissues was analyzed using The Cancer Genome Atlas (TCGA) dataset. Bioinformatics approaches, cellular models, and clinical tissue microarrays were employed to investigate the functional role of IL36RN, its interactions within the TME, and its prognostic significance.

**Results:**

IL36RN expression was markedly upregulated in GC tissues and associated with unfavorable survival outcomes. Functional assays demonstrated that IL36RN silencing suppressed GC cell proliferation and invasion. Elevated IL36RN expression correlated with enhanced CD8^+^ T cell infiltration in the TME and served as an independent prognostic indicator in GC.

**Conclusions:**

IL36RN represents a potential prognostic biomarker and therapeutic target in GC, offering novel avenues for precision oncology and immunotherapeutic intervention.

## 1 Background

Gastric cancer (GC) remains a major contributor to cancer‐related mortality worldwide, necessitating the development of more effective therapeutic strategies [1]. Immune checkpoint blockade (ICB) has emerged as a promising immunotherapy for GC and other malignancies by modulating the tumor microenvironment (TME) [2]. Given the pivotal role of the TME in tumor progression and therapeutic response, targeting its components is essential for enhancing the precision and efficacy of cancer treatments [3]. However, the heterogeneous responses observed among patients with GC underscore the need for a deeper understanding of TME dynamics to improve immunotherapy outcomes [4, 5].

Despite advancements in cancer research, the development of effective therapies remains hindered by the lack of preclinical models that accurately recapitulate the TME [6]. Although targeted immunotherapies have demonstrated clinical potential, their efficacy is restricted to a subset of patients, and predicting treatment responses remains a formidable challenge [7, 8, 9]. This limitation largely stems from the intricate interplay between tumor neoantigens and immune cells within the TME, where the spatial organization and functional heterogeneity of immune cell populations exert a profound influence on therapeutic success [10, 11]. A comprehensive understanding of tumor‐immune interactions and the regulatory mechanisms within the TME is therefore crucial for advancing personalized cancer therapies [12].

Cytokines, particularly interleukins (ILs), serve as key regulators of antitumor immunity, orchestrating communication between immune, stromal, and cancer cells within the TME [13]. Aberrant cytokine signaling can drive tumor progression, whereas precise modulation of these pathways holds promise for enhancing immune surveillance and antitumor responses [14, 15]. Among these, IL‐36 cytokines—members of the IL‐1 family—have garnered attention for their role in inflammation and immune cell recruitment [16, 17]. The interleukin‐36 receptor antagonist (IL36RN), also known as IL1F5, is a key immunomodulator within this family. While IL36RN has been extensively studied in autoimmune disorders such as psoriasis and inflammatory bowel disease, its role in cancer immunity remains poorly characterized [18, 19, 20]. Notably, the impact of IL36RN on tumor‐infiltrating lymphocytes and its relevance to GC progression and immunotherapy response remain unexplored.

This study aims to bridge this critical knowledge gap by elucidating the role of IL36RN in GC. It is hypothesized that IL36RN modulates the TME and influences GC progression, positioning it as a potential therapeutic target. The specific objectives of this investigation are to characterize IL36RN expression in GC tissues and its association with patient prognosis, delineate its functional role in GC cell proliferation and invasion, and examine its relationship with immune cell infiltration in the TME. By defining the role of IL36RN in GC, this study aims to provide novel insights into TME regulation and facilitate the development of targeted immunotherapies for patients with GC.

## 2 Materials and Methods

### 2.1 TCGA Database Data Retrieval

Data from the TCGA database were utilized in this study, encompassing transcriptomic and clinical information from 375 GC samples retrieved from the Genome Data Commons (GDC) (https://portal.gdc.cancer.gov/).

### 2.2 Validation Cohort

The validation cohort comprised 336 GC tissue samples and 67 normal gastric mucosal tissue samples obtained from the Affiliated Hospital of Nantong University (AHNU). Clinicopathological data were assessed, and patients with incomplete survival information or unknown prognostic status were excluded. Additionally, patients who had undergone preoperative chemotherapy or radiotherapy or had a history of other malignancies were not included in the study. Tissue microarrays (TMA) were constructed using the manual Tissue Microarrayer System Quick Ray (UT06, UNITMA, Korea). Specifically, core tissue samples (2 mm in diameter) were extracted from approximately 70 separate formalin‐fixed paraffin‐embedded (FFPE) blocks and re‐embedded into a new paraffin block. Sections of 4 μm thickness were subsequently sliced and mounted onto SuperFrost‐charged glass slides for TMA analysis. Ethical approval was granted by the AHNU Ethics Committee (No. 2018‐K020), and all procedures were conducted in accordance with the principles outlined in the Helsinki Declaration.

### 2.3 Cell Lines

Four human GC cell lines—MKN45, MKN1, HGC‐27, and AGS—were obtained from Cobioer (Nanjing, China) and maintained in complete media consisting of 90% RPMI 1640 medium (Thermo Fisher, New York, USA), 10% fetal bovine serum (FBS, Gibco, California, USA), and 1% penicillin–streptomycin (Gibco, California, USA) under standard culture conditions (37°C, 5% CO_2_). To ensure consistent growth characteristics, all cell lines were passaged within 10–15 passages. Routine mycoplasma testing was conducted using the MycoBlue Mycoplasma Detector (D101, Vazyme, Nanjing, China) to confirm the absence of mycoplasma contamination.

### 2.4 Transfection

Upon reaching 70%–80% confluency, plasmid transfection was performed using LipoFiter 3.0 (Hanbio, Shanghai, China) according to the manufacturer's protocol. Two EP tubes were each filled with 250 μL of basal culture medium. One tube contained 5 μg of plasmid DNA, while the other contained 10 μL of LipoFiter 3.0 transfection reagent. Both tubes were incubated separately for 5 min, after which their contents were combined and incubated for an additional 20 min. The resulting mixture was then added dropwise to the wells of a 6‐well plate. Following a 6‐h incubation, the medium was replaced with fresh culture media, and the cells were further incubated as per the experimental protocol. Two control groups were established: a non‐targeting shRNA control (sh‐NC) and an untreated control (Con). The IL36RN shRNA sequence used for transfection was 5'‐GCAGGGAAGGTCATTAAAGGT‐3'.

### 2.5 Western Blotting

Total protein was extracted from cells using pre‐chilled lysis buffer, mixed with 5× loading buffer, resolved by SDS‐PAGE, and subsequently transferred onto PVDF membranes. The membranes were blocked with 5% nonfat milk, incubated overnight at 4°C with primary antibodies targeting IL36RN (1:1000, 60,298‐1‐Ig, Proteintech, Wuhan, China), followed by treatment with HRP‐conjugated secondary antibodies. Protein detection was performed using an enhanced chemiluminescence (ECL) reagent and visualized on an ECL imaging system (Tanon, Shanghai, China).

### 2.6 Transwell Invasion Assay

For the invasion assay, BD Matrigel was pre‐coated onto the upper chamber and incubated overnight. The lower chamber was supplemented with 700 μL of RPMI 1640 medium containing 10% FBS, while transfected cells were seeded into the upper chamber. After a 24‐h incubation, invasive cells on the lower membrane surface were fixed with 4% paraformaldehyde, stained with crystal violet, and subsequently quantified using ImageJ software.

### 2.7 Cell Proliferation Assessment

Cell proliferation was evaluated using a CCK‐8 assay (Meilunbio, Dalian, China). Transfected cells were seeded into 96‐well plates and incubated at 37°C for 0, 24, 48, or 72 h. After the respective incubation periods, CCK‐8 reagent was added and incubated for 2 h, followed by absorbance measurement at 450 nm. Growth curves were generated to assess proliferation rates.

### 2.8 Multiplex Immunohistochemistry

Multiplex immunohistochemistry was performed on TMAs derived from the AHNU cohort. Serial TMA sections were prepared, and multiplex immunofluorescence staining was conducted according to established protocols [21]. Antigen retrieval was performed using AR6 buffer (AR900250ML, AKOYA, Boston, USA) and AR9 buffer (AR600250ML, AKOYA, Boston, USA). Sequential rounds of staining were carried out, followed by nuclear counterstaining with DAPI (F6057, Sigma, Saint Louis, USA), and the TMAs were subsequently mounted. The following primary antibodies were utilized: anti‐IL36RN (1:1000, PA5‐72779, Thermo Fisher, New York, USA), anti‐CD4 (1:600, ab133616, Abcam, Cambridge, UK), anti‐CD3 (1:600, 85061S, Cell Signaling Technology, Massachusetts, USA), anti‐CD66b (1:500, ARG66287, Arigo, Shanghai, China), anti‐CD20 (1:600, ab78237, Abcam, Cambridge, UK), anti‐CD68 (1:600, 76437S, Cell Signaling Technology, Massachusetts, USA), anti‐CTLA4 (1:400, orb527271, Biorbyt, Cambridge, UK), anti‐PD‐1 (1:400, 86163S, Cell Signaling Technology, Massachusetts, USA), anti‐PD‐L1 (1:400, 13684S, Cell Signaling Technology, Massachusetts, USA), and anti‐cytokeratin (1:1000, orb69073, Biorbyt, Cambridge, UK).

The stained TMAs were scanned using the AKOYA automated imaging system, and protein expression was quantified through a semiquantitative scoring method using Inform image analysis software. For statistical analysis, patients were stratified into two groups (low/no expression vs. high expression) based on a predefined cutoff value. The optimal threshold for protein expression was determined using X‐tile software (Yale University, Connecticut, USA), ensuring significant stratification of overall survival (OS). Specifically, samples exhibiting expression levels at or below the cutoff were classified as low/no expression, whereas those exceeding the threshold were categorized as high expression. Protein expression assessment was conducted independently of patient outcome data to eliminate bias.

### 2.9 Isolation and Culture of CD8+ T Cells

CD8^+^ T cells were isolated and cultured from human peripheral blood using a combination of density gradient centrifugation and magnetic bead‐based sorting, followed by activation and expansion in a complete medium. Peripheral blood mononuclear cells (PBMCs) were separated using Ficoll (FMS‐900014, FcMACS, Nanjing, China) via density gradient centrifugation. Peripheral blood was diluted 1:1 with phosphate‐buffered saline (PBS) and carefully layered onto the separation solution. Following centrifugation at 800×*g* for 20 min at room temperature, the PBMC layer was collected from the plasma–separation solution interface and washed twice with PBS before resuspension in a complete medium for subsequent processing. CD8^+^ T cells were further enriched using the RWD Human CD8^+^ Cell Isolation Kit (K1203‐10, RWD, Shenzhen, China), which employs a magnetic bead‐based negative selection strategy. PBMCs were resuspended in the isolation buffer at a density of 1 × 10^8^ cells/mL and incubated with magnetic beads at 4°C for 15 min, with gentle mixing every 5 min. After incubation, the cell suspension was exposed to a magnetic field, allowing the separation of unlabeled CD8^+^ T cells from magnetically labeled non‐target cells. The supernatant containing the purified CD8^+^ T cell fraction was collected and washed twice with PBS to eliminate any residual beads. The isolated CD8^+^ T cells were cultured in a complete medium consisting of 90% RPMI 1640 (Thermo Fisher, New York, USA), 10% fetal bovine serum (FBS; Gibco, California, USA), and 1% penicillin–streptomycin (Gibco, California, USA). Cells were maintained under standard culture conditions (37°C, 5% CO_2_, humidified atmosphere). To induce activation and proliferation, recombinant human IL‐2 (PB180634, Procell, Wuhan, China) was added at a final concentration of 100 U/mL. On the following day, activated CD8^+^ T cells were transfected with the IL36RN shRNA plasmid. Two control groups were included: an untreated group (Con) and a control shRNA‐transfected group (sh‐NC). Transfection was carried out according to the manufacturer's protocol, and cells were cultured for an additional 48 h. Following this period, cell viability was assessed using standard methodologies to evaluate the impact of IL36RN shRNA transfection on CD8^+^ T cell activity.

### 2.10 Data Analysis

Pearson's *χ* ^2^ test was employed to assess the association between IL36RN protein expression and clinicopathological characteristics. Continuous variables were analyzed using the Mann–Whitney *U* test, while categorical variables were evaluated with the *χ* ^2^ test. Comparisons between groups were conducted using Student's *t*‐test or one‐way ANOVA, as appropriate. Statistical analyses were performed using SPSS 22.0, with optimal cutoff values determined via X‐tile software. The Kruskal‐Wallis test was applied to compare gene expression levels, and Kaplan–Meier survival analysis was used to evaluate overall survival, with validation performed using publicly available datasets. All experiments were conducted with samples from at least three independent biological replicates to ensure reproducibility.

## 3 Results

### 3.1 IL36RN mRNA Expression in Patients With GC

**FIGURE 1:**
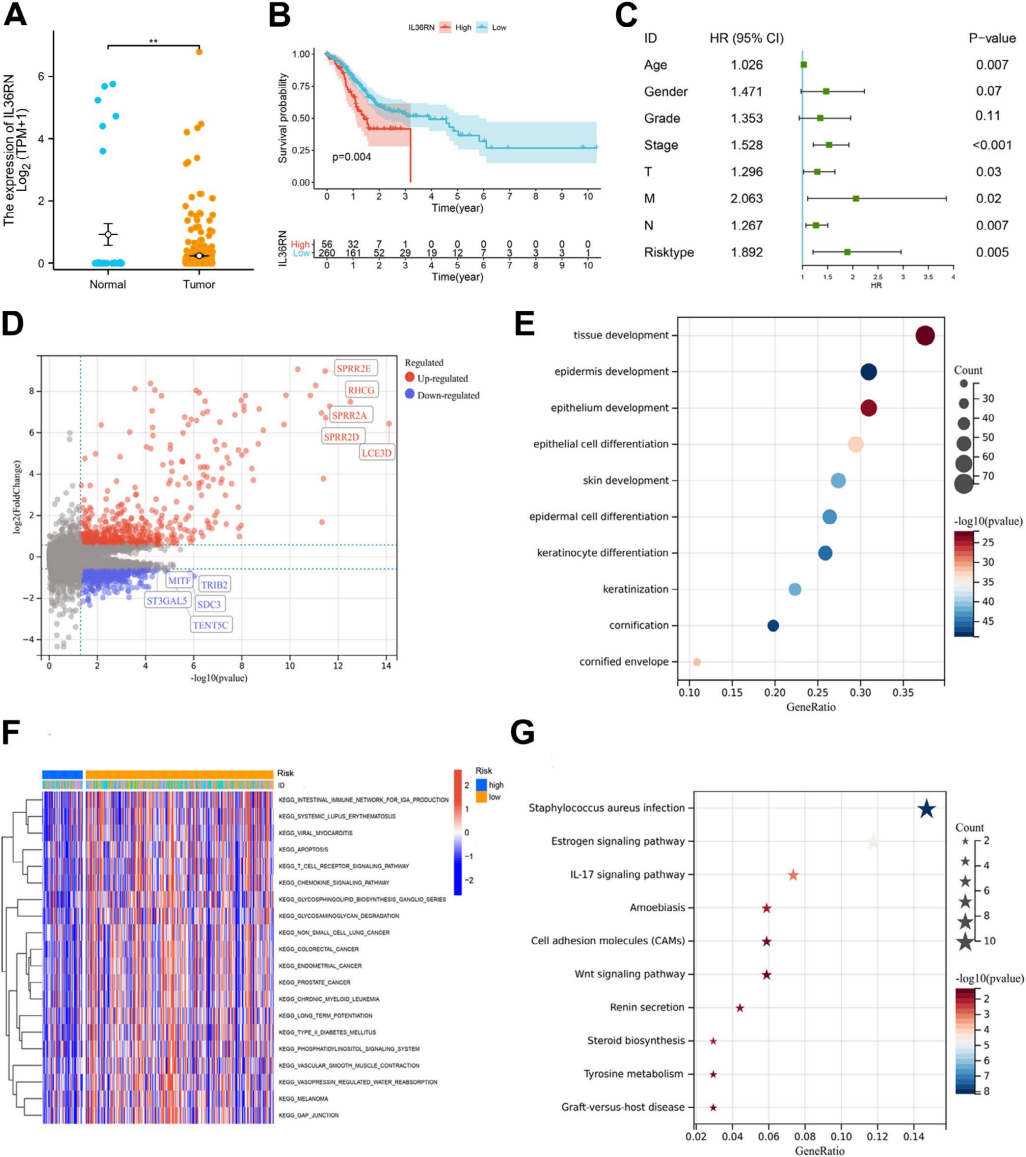
IL36RN mRNA expression is associated with prognosis and multiple signaling pathways in gastric cancer. (A) IL36RN mRNA expression levels in gastric cancer and adjacent non‐tumor tissues in the TCGA cohort. (B) Kaplan–Meier survival analysis illustrating the association between IL36RN mRNA expression and overall survival in patients with gastric cancer from the TCGA cohort. (C) Forest plot of univariate Cox regression analysis for overall survival (OS) in the TCGA cohort. (D) Volcano plot displaying differentially expressed genes (DEGs) stratified by IL36RN expression. Red dots indicate upregulated genes, blue dots denote downregulated genes, and gray dots represent genes without statistically significant differences. (E) Dot plot summarizing Gene Ontology (GO) enrichment analysis of significant DEGs. (F) Heatmap showing the top 20 pathways identified through Gene Set Variation Analysis (GSVA). (G) Dot plot illustrating Kyoto Encyclopedia of Genes and Genomes (KEGG) pathway enrichment analysis of significant DEGs.

IL36RN mRNA expression was significantly elevated in GC tissues compared to adjacent non‐tumor tissues. Patients with high IL36RN mRNA expression exhibited poorer survival outcomes than those with low expression levels (*p* < 0.01, Figure 1A,B). Cox regression analysis identified age, T stage, N stage, M stage, TNM stage, and risk classification as independent prognostic factors for GC (HR > 1, *p* < 0.05, Figure 1C).

To further elucidate the functional role of IL36RN, bioinformatics analyses were conducted to investigate IL36RN‐associated signaling pathways. Differentially expressed genes (DEGs) were identified based on IL36RN expression levels and visualized using volcano plots (Figure 1D). Gene Ontology (GO) and Kyoto Encyclopedia of Genes and Genomes (KEGG) pathway enrichment analyses were performed on the significant DEGs. GO analysis revealed enrichment in cancer‐related biological processes, including tissue development, epidermal development, and epithelial cell differentiation (Figure 1E). KEGG pathway analysis highlighted key pathways such as *Staphylococcus aureus* infection, estrogen signaling, IL‐17 signaling, amoebiasis, and cell adhesion molecule pathways (Figure 1G). Additionally, gene set variation analysis (GSVA) identified pathways potentially associated with IL36RN, including the IgA‐producing intestinal immune network, systemic lupus erythematosus, viral myocarditis, apoptosis, T‐cell receptor signaling, and chemokine signaling pathways (Figure 1F). These results suggest that IL36RN may play a role in GC prognosis and is involved in multiple oncogenic and immune‐related biological processes, underscoring its potential complexity in GC progression.

### 3.2 IL36RN Interference Weakened GC Cell Invasion and Proliferation

**FIGURE 2:**
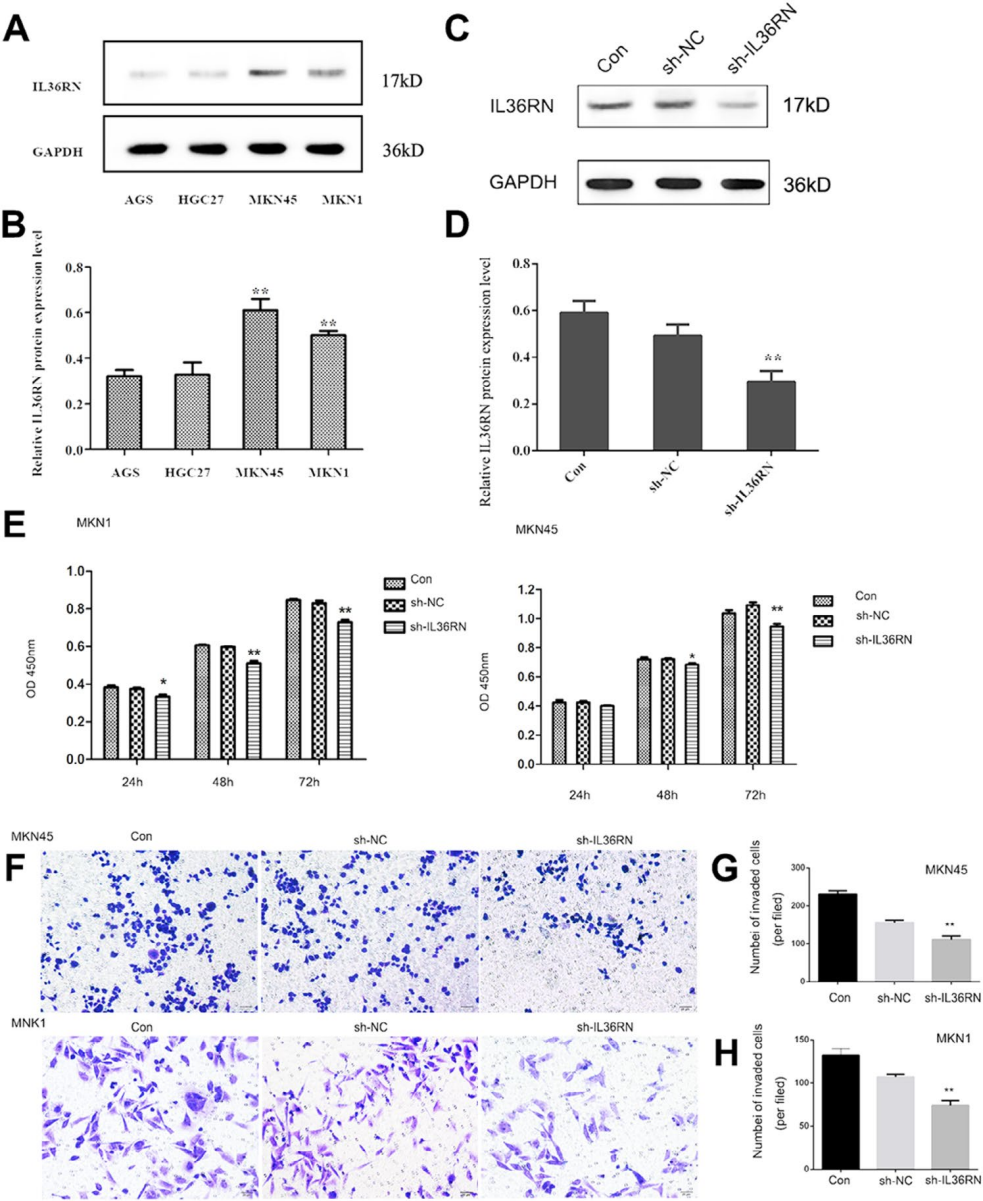
IL36RN downregulation inhibits gastric cancer cell proliferation and invasion. (A, B) IL36RN protein expression levels in four gastric cancer cell lines, accompanied by densitometric analysis of gray values. (C, D) Western blot analysis demonstrating the effect of shRNA‐mediated IL36RN knockdown on IL36RN protein levels in gastric cancer cells. (E) CCK‐8 assay results indicating the impact of IL36RN knockdown on the proliferation of MKN1 and MKN45 cells. (F–H) Representative Transwell invasion assay images showing reduced invasive capacity in MKN1 and MKN45 cells following IL36RN knockdown compared to the control group.

To investigate the functional impact of IL36RN in GC, IL36RN knockdown was achieved using shRNA. Initially, IL36RN protein expression was evaluated in four GC cell lines (AGS, HGC‐27, MKN45, and MKN1). MKN45 and MKN1 exhibited higher IL36RN protein expression levels than AGS and HGC‐27 (Figure 2A,B). Transfection with shRNA plasmids effectively reduced IL36RN protein levels, as confirmed by Western blot analysis, with a significant reduction observed in the knockdown group compared to the control group (Figure 2C,D), thereby validating the successful establishment of IL36RN‐knockdown cell lines.

Functional assays demonstrated that IL36RN downregulation markedly inhibited GC cell proliferation (Figure 2E). Additionally, the invasive potential of MKN1 and MKN45 cells was significantly attenuated following IL36RN knockdown (Figure 2F–H). Collectively, these results indicate that IL36RN downregulation substantially suppresses the proliferation and invasion of MKN1 and MKN45 GC cells, highlighting its potential regulatory significance in GC progression.

### 3.3 IL36RN Protein Expression in GC Tissues and Benign Stomach Tissues

**FIGURE 3:**
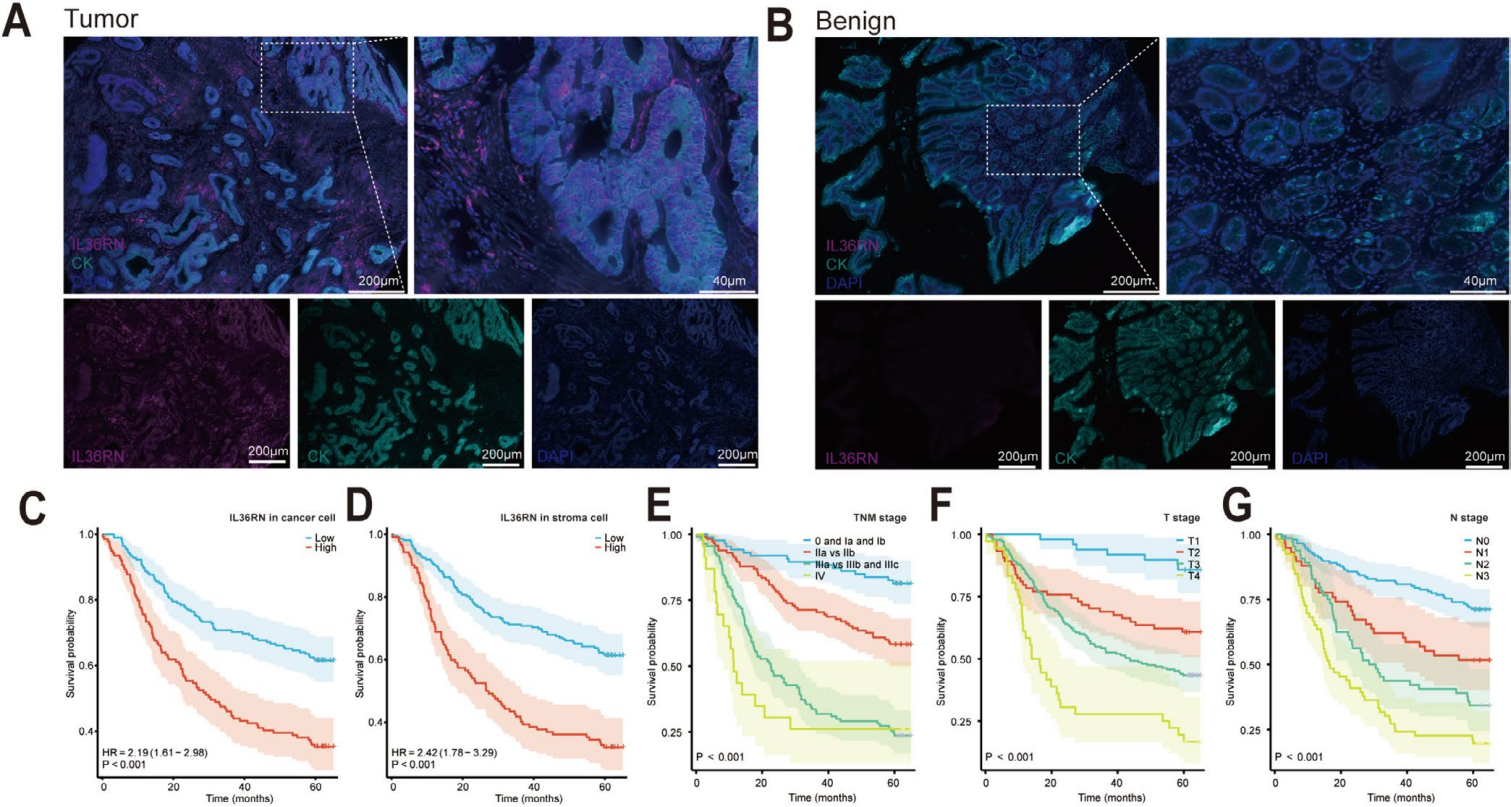
IL36RN protein expression in gastric cancer tissues and its prognostic significance. (A, B) Immunofluorescence images depicting IL36RN protein expression and localization in gastric cancer tissues versus benign gastric tissues. (C–G) Kaplan–Meier survival curves illustrating the relationship between IL36RN protein expression in cancerous and stromal regions and patient survival outcomes, stratified by TNM stage, T stage, and N stage.

Analysis of the TCGA dataset highlighted the potential clinical significance of IL36RN; however, given that mRNA and protein expression levels do not always exhibit a linear correlation, further validation was conducted by assessing IL36RN protein expression in clinical samples using GC TMAs and multiplex immunofluorescence staining. The results demonstrated significantly higher IL36RN protein expression in GC tissues compared to benign gastric tissues (Figure 3A,B). Additionally, an imaging‐based classification system was employed to delineate cancer nests and stromal regions, allowing for a compartmentalized evaluation of IL36RN expression. The observed expression patterns were consistent with those derived from the TCGA dataset.

### 3.4 Correlation Between IL36RN Protein Expression and GC Clinical Features

To determine the optimal cut‐off values for IL36RN protein expression in cancer and stromal cells, X‐tile software was used. The cut‐off values were defined as 56.0 for cancer cells and 22.0 for stromal cells, with expression levels at or below these thresholds classified as low expression, while levels exceeding the thresholds were categorized as high expression. The association between IL36RN expression and clinicopathological characteristics was further analyzed (Table 1), revealing a significant correlation between IL36RN expression in cancer cells and tumor stage (*p* < 0.05). Similarly, IL36RN expression in stromal regions exhibited strong associations with TNM stage (*p* < 0.001), T stage (*p* = 0.016), N stage (*p* = 0.002), and M stage (*p* = 0.007). These results indicate that elevated IL36RN protein expression is strongly linked to advanced GC TNM staging.

**TABLE 1:** Relationship between the expression of IL36RN protein and clinical characteristics.

Characteristic	*n*	High expression of IL36RN in cancer cell (%)	Pearson *χ* ^2^	*p*	High expression of IL36RN in stroma cell (%)	Pearson *χ* ^2^	*p*
Total	336	141 (42.09)			124 (36.90)		
Gender			0.990	0.320		0.458	0.499
Male	243	106 (43.62)			87 (35.80)		
Female	93	35 (37.63)			37 (39.78)		
Age			2.213	0.137		0.852	0.356
< 60	130	48 (36.92)			44 (33.85)		
≥ 60	206	93 (45.15)			80 (38.83)		
Laurén classification			0.101	0.951		4.129	0.127
Intestinal type	312	131 (41.99)			118 (37.82)		
Diffuse type	21	9 (42.86)			4 (19.05)		
Mixed type	3	1 (33.33)			2 (66.67)		
Differentiation (Tubular adenocarcinoma)			3.262	0.196		2.758	0.252
Well	14	6 (42.86)			3 (21.43)		
Middle	97	48 (49.48)			32 (32.99)		
Poor	225	87 (38.67)			89 (39.56)		
T			5.359	0.147		10.349	0.016*
T1	49	20 (40.82)			12 (24.49)		
T2	75	31 (41.33)			24 (32.00)		
T3	177	69 (38.98)			68 (38.42)		
T4	35	21 (60.00)			20 (57.14)		
N			14.242	0.003*		14.508	0.002*
N0	147	49 (33.33)			39 (26.53)		
N1	58	21 (36.21)			22 (37.93)		
N2	64	32 (50.00)			33 (51.56)		
N3	67	39 (58.21)			30 (44.78)		
M			6.476	0.011*		7.272	0.007*
M0	312	125 (40.06)			109 (34.94)		
M1	24	16 (66.67)			15 (62.50)		
TNM stage			17.771	< 0.001*		26.178	< 0.001*
0 and Ia and Ib	87	30 (34.48)			21 (24.14)		
IIa versus IIb	115	37 (32.17)			32 (27.83)		
IIIa versus IIIb and IIIc	110	58 (52.73)			56 (50.91)		
IV	24	16 (66.67)			15 (62.50)		
Preoperative CA199, U/mL			1.533	0.465		1.421	0.491
≤ 37	154	63 (40.91)			62 (40.26)		
> 37	25	8 (32.00)			9 (36.00)		
Unkown	157	70			53		
Preoperative CEA, ng/mL			3.425	0.180		0.738	0.691
≤ 5	146	53 (36.30)			53 (36.30)		
> 5	42	19 (45.24)			18 (42.86)		
Unkown	148	69			53		
HER‐2 status			6.417	0.170		6.159	0.188
0	255	99 (38.82)			92 (36.08)		
1+	26	14 (53.85)			12 (46.15)		
2+	29	14 (48.28)			12 (41.38)		
3+	24	12 (50.00)			6 (25.00)		
Unkown	2	2			2		

* *p* < 0.05.

### 3.5 Elevated IL36RN Expression in GC Tissues Is Associated With Decreased Patient Survival and Indicates Poor Prognosis

Multivariate Cox regression analysis identified IL36RN expression in both cancer and stromal cells, along with T, N, and TNM staging, as independent prognostic factors (Table 2, HR > 1, *p* < 0.05). Notably, increased IL36RN expression in GC tissues correlated with reduced overall survival, underscoring its role as an independent prognostic marker. Survival analysis revealed that both high‐expression groups (cancer cell and stromal cell) exhibited significantly worse survival outcomes compared to their respective low‐expression groups (Figure 3C,D *p* < 0.001). Additional factors associated with diminished patient survival are depicted in Figure 3E–G.

**TABLE 2:** Univariate and multivariable analysis of prognostic factors for 5‐year survival in gastric cancer.

	Univariate analysis				Multivariate analysis (adjusted for age)			
	HR	*p* > |z|	95% CI		HR	*p* > |z|	95% CI	
IL36RN expression in cancer cell	2.194	< 0.001*	1.614	2.984	1.487	0.042*	1.015	2.179
High versus low and none								
IL36RN expression in stroma cell	2.417	< 0.001*	1.779	3.285	1.489	0.035*	1.029	2.156
High versus low and none								
Gender	0.852	0.344	0.611	1.188				
Male versus Female								
Age (years)	1.472	0.021*	1.061	2.041	1.280	0.146	0.918	1.784
≤ 60 versus > 60								
Laurén classification	1.351	0.201	0.852	2.143				
Intestinal type versus Diffuse type versus Mixed type								
Differentiation (Tubular adenocarcinoma)	1.360	0.037*	1.019	1.813	1.122	0.485	0.812	1.550
Well and Middle versus poor								
TNM stage	2.224	< 0.001*	1.865	2.651	1.499	0.014*	1.087	2.068
0 and Ia and Ib versus IIa versus IIb versus IIIa versus IIIb and IIIc versus IV								
T	2.043	< 0.001*	1.659	2.517	1.340	0.037*	1.017	1.765
T1 versus T2 versus T3								
N	1.705	< 0.001*	1.498	1.940	1.240	0.020*	1.034	1.487
N0 versus N1 versus N2 versus N3								
M	2.781	< 0.001*	1.680	4.604				
M0 versus M1a and M1b								
Preoperative CA199, U/mL	1.015	0.848	0.868	1.188				
≤ 37 versus > 37								
Preoperative CEA, ng/mL	1.104	0.228	0.940	1.296				
≤ 5 versus > 5								
Her‐2	1.065	0.410	0.916	1.239				
0 versus 1+ versus 2+ versus 3+								

* *p* < 0.05.

### 3.6 IL36RN Is Associated With Tumor‐Infiltrating Immune Cells in GC TME

**FIGURE 4:**
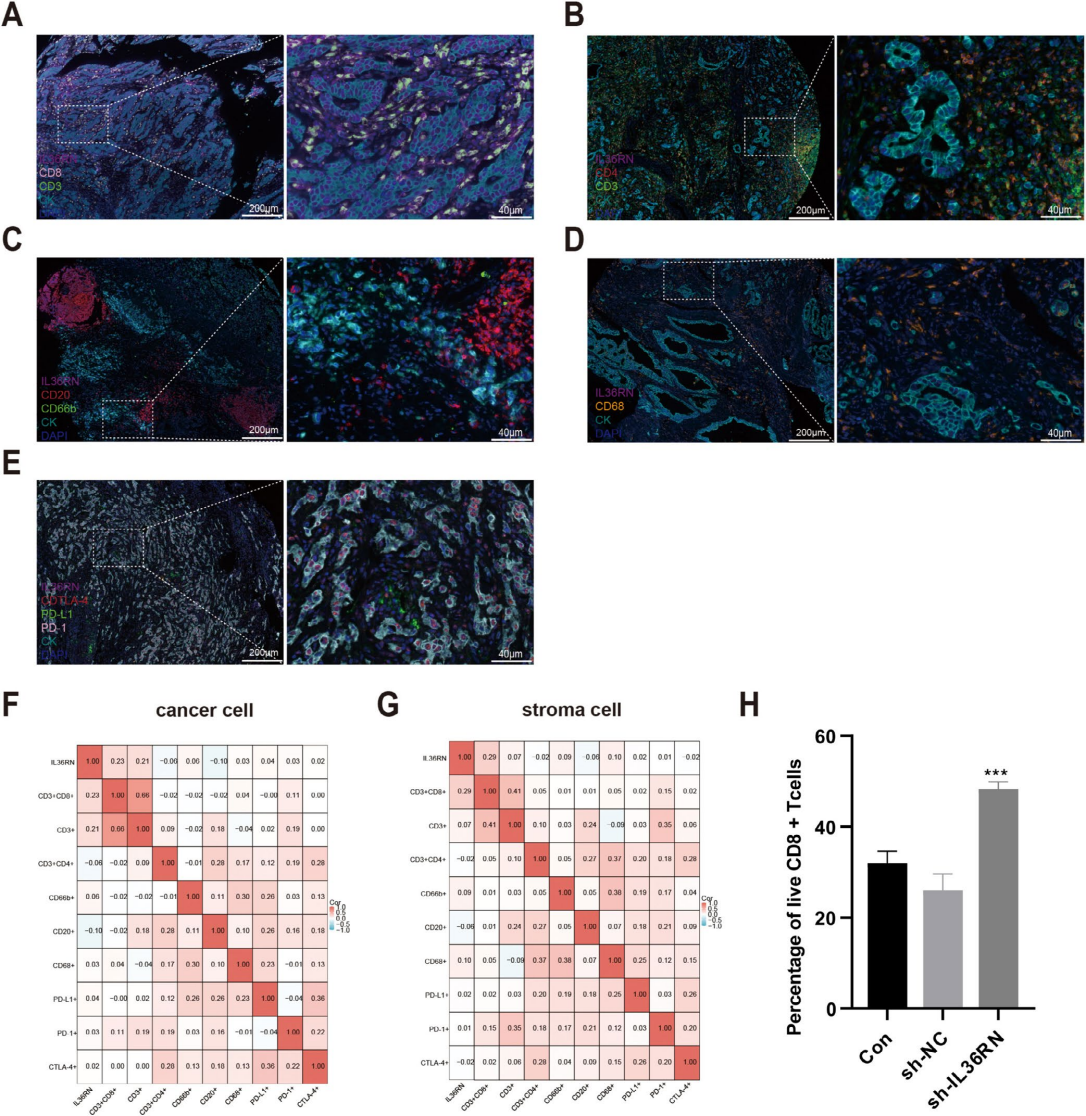
IL36RN expression and its correlation with tumor‐infiltrating immune cells in gastric cancer. (A) Representative multiplex immunofluorescence images displaying IL36RN expression and CD8^+^ T cell infiltration in gastric cancer tissues. (B) Representative multiplex immunofluorescence images showing IL36RN expression alongside CD4^+^ T cell abundance in gastric cancer tissues. (C) Representative multiplex immunofluorescence images illustrating IL36RN expression in relation to B cell and neutrophil infiltration in gastric cancer tissues. (D) Representative multiplex immunofluorescence images depicting IL36RN expression and macrophage infiltration in gastric cancer tissues. (E) Representative multiplex immunofluorescence images showing IL36RN expression alongside immune checkpoint markers (e.g., PD‐1, PD‐L1) in gastric cancer tissues. (F, G) Positive correlation between IL36RN protein expression and various immune cell populations in both cancerous and stromal regions of gastric cancer tissues. (H) CD8^+^ T cell viability following IL36RN knockdown. Data are presented as mean ± SEM from three independent experiments. ***p < 0.01 versus control.

IL‐36 cytokines, secreted by various immune cells, drive the production of proinflammatory mediators such as IL‐1 and IL‐23, thereby shaping immune responses. To elucidate the role of IL36RN in GC, its association with tumor‐infiltrating immune cells (TIICs) was examined using multiplex immunofluorescence to identify immune cell subtypes within GC tissues. The analysis revealed that TIICs predominantly localized within stromal regions (Figure 4A–E). A significant positive correlation was observed between IL36RN expression and CD8^+^ T cell infiltration in both the cancer nest (*R* = 0.23, *p* < 0.05; Figure 4F) and stromal compartment (*R* = 0.29, *p* < 0.05; Figure 4G), suggesting that IL36RN may influence CD8^+^ T cell dynamics in the TME and could serve as a potential therapeutic target in GC. To further investigate the functional impact of IL36RN on CD8^+^ T cells, PBMCs were isolated from human peripheral blood and subjected to magnetic bead sorting for CD8^+^ T cell enrichment. IL36RN expression was then silenced in these cells, and viability was assessed. The results demonstrated that IL36RN knockdown significantly enhanced CD8^+^ T cell viability compared to the control group (Figure 4H). These results indicate that IL36RN modulates the tumor immune microenvironment by suppressing CD8^+^ T cell cytotoxicity, potentially facilitating immune evasion in GC. This observation aligns with earlier findings, demonstrating that elevated IL36RN expression correlates with poorer patient prognosis.

## 4 Discussion

IL36RN, an immunomodulatory protein, serves as a critical regulator of the IL‐36 signaling pathway. Mutations or deletions in IL36RN result in dysregulated IL‐36 activity, triggering excessive immune responses and contributing to severe manifestations of generalized pustular psoriasis [22]. While most research on IL36RN has focused on its role in autoimmune diseases, particularly psoriasis [22], the IL‐36 cytokine family has also been explored as a potential therapeutic target for various inflammatory conditions [23, 24]. At the mRNA level, IL36RN has been implicated in tumorigenesis and cancer progression. The present study demonstrated that elevated IL36RN mRNA expression correlates with poorer survival outcomes in patients with GC, with validation across multiple independent datasets further reinforcing its potential as a prognostic biomarker in GC.

IL36RN proteins are secreted by various immune cell types, including T lymphocytes, B lymphocytes, and macrophages/monocytes [25]. Among IL‐36 family members, IL‐36β has been shown to induce M2‐like macrophage polarization and activate Langerhans cells. Notably, IL36RN is predominantly expressed in naïve T cells, where it facilitates their differentiation into Th1 and Th17 subtypes, thereby enhancing inflammatory cytokine production [17]. Additionally, IL36RN exerts stimulatory effects on keratinocytes, fibroblasts, and epithelial cells, indicating its functional relevance in both immune and non‐immune cellular contexts [18].

The present study identified significant associations between IL36RN expression and multiple immune cell types in both cancerous and stromal compartments, with the strongest correlation observed with CD8^+^ T cells. These findings suggest that IL36RN plays a pivotal role in shaping the immune microenvironment of GC, potentially modulating anti‐tumor immune responses through its interactions with CD8^+^ T cells.

IL‐36γ has been shown to enhance the activation and expansion of CD8^+^ T cells while significantly upregulating IFN‐γ and IL‐2 expression, thereby exerting potent antitumor effects [26, 27]. However, IL‐36Ra functions as a physiological antagonist of IL‐36γ, potentially dampening IL‐36γ‐induced immune activation and modulating immune homeostasis within the tumor microenvironment (TME) [28]. As a pro‐inflammatory cytokine, IL‐36γ binds to the IL‐36 receptor (IL‐36R), facilitating the recruitment of IL‐1 receptor accessory protein (IL‐1RAcP) [29, 30]. This interaction activates NF‐κB and MAPK signaling cascades, leading to the upregulation of pro‐inflammatory gene expression and amplifying inflammatory responses [29, 30]. Conversely, IL‐36Ra, encoded by IL36RN, competes with IL‐36γ for IL‐36R binding, preventing IL‐1RAcP recruitment and effectively blocking IL‐36R downstream signaling. This antagonistic mechanism plays a pivotal role in modulating IL‐36‐driven inflammatory responses and immune regulation [31]. In non‐small cell lung cancer (NSCLC) and colorectal cancer, IL‐36γ and IL‐36Ra differentially regulate tumorigenesis and tumor progression by influencing extracellular matrix remodeling and the Wnt signaling pathway [32]. The NF‐κB and MAPK pathways, activated by IL‐36γ, are integral to inflammatory and immune responses, while IL‐36Ra exerts an immunosuppressive effect by inhibiting these pathways. This antagonistic interplay is essential for maintaining immune equilibrium within the TME [28, 33]. In summary, IL‐36γ and IL36RN (IL‐36Ra) exhibit an antagonistic interaction within signaling pathways, playing a critical role in regulating inflammatory responses and tumor immunity. Given these findings, IL36RN functions as an IL‐36γ antagonist, potentially suppressing CD8^+^ T cell activity and promoting immune evasion within the tumor microenvironment.

By inhibiting the activity of IL‐36 family members, IL36RN regulates NF‐κB and MAPK signaling pathways and modulates adaptive immunity [18, 34, 35, 36, 37]. Understanding specific immune dysfunctions that arise during tumor progression remains a critical area of research [37]. Further investigation is warranted to determine whether IL36RN mediates immune suppression by attenuating NF‐κB and MAPK signaling in GC, thereby impairing antitumor immune responses. Targeting IL36RN may represent a novel therapeutic strategy for personalized treatment, offering potential avenues to enhance immune activation and improve patient outcomes.

IL36RN expression in both cancerous and stromal regions exhibits a significant correlation with immune cell infiltration in GC, positioning it as a potential prognostic marker. Its association with CD8^+^ T cell levels may further refine prognosis prediction accuracy.

Despite these findings, several limitations must be acknowledged. The reliance on retrospective data and bioinformatics analyses restricts the ability to establish causal relationships. Additionally, the absence of long‐term follow‐up data limits the assessment of IL36RN's prognostic significance over extended periods. Moreover, the precise mechanisms through which IL36RN modulates immune cell activity in GC remain unclear. Future studies should prioritize functional investigations to delineate the direct effects of IL36RN on immune‐related signaling pathways, such as NF‐κB and MAPK, which are key regulatory axes of IL‐36 family members.

The observed correlation between IL36RN expression and immune cell infiltration, particularly CD8^+^ T cells, underscores its potential role as a prognostic biomarker in GC. Integrating IL36RN expression levels into prognostic models may enhance predictive accuracy and facilitate the development of personalized treatment strategies. Furthermore, therapeutic interventions targeting IL36RN could provide a novel approach to modulate the tumor immune microenvironment, potentially improving clinical outcomes for patients with GC.

In conclusion, this study highlights IL36RN as a promising prognostic marker and therapeutic target in GC. Future research should focus on elucidating its mechanistic role in tumor immunity and evaluating its clinical applicability in GC treatment.

## Author Contributions

**Xiaojing Zhang:** conceptualization (equal), data curation (equal), formal analysis (lead), funding acquisition (equal), methodology (lead), software (lead), writing – original draft (lead). **Sutian Jiang:** conceptualization (equal), data curation (equal), methodology (equal), software (equal), writing – original draft (equal). **Hang Yin:** formal analysis (equal), investigation (equal), methodology (equal). **Hui Zhang:** methodology (equal), resources (equal), software (equal). **Lei Yang:** formal analysis (equal), software (equal), software (equal), validation (equal), validation (equal). **Pingping Sun:** conceptualization (equal), investigation (equal). **Xiaoling Kuai:** funding acquisition (equal), resources (equal). **Chen Chen:** funding acquisition (equal), investigation (equal), methodology (equal), project administration (equal), writing – review and editing (equal). **Jianfei Huang:** conceptualization (lead), data curation (lead), funding acquisition (lead), investigation (lead).

## Ethics Statement

Ethical approval was obtained from the Affiliated Hospital of Nantong University Ethics Committee (No. 2018‐K020).

## Conflicts of Interest

The authors declare no conflicts of interest.

## Data Availability

The datasets generated during and/or analyzed during the current study are not publicly available due [The data involves private patient information.] but are available from the corresponding author on reasonable request.
